# Preparation of Ampicillin Surface Molecularly Imprinted Polymers for Its Selective Recognition of Ampicillin in Eggs Samples

**DOI:** 10.1155/2018/5897381

**Published:** 2018-11-15

**Authors:** Yang Tian, Yue Wang, Shanshan Wu, Zhian Sun, Bolin Gong

**Affiliations:** College of Chemistry and Chemical Engineering, North Minzu University, Yinchuan, 750021, China

## Abstract

Surface-imprinted polymers (MIPs) microspheres with the ability to specifically recognize water-soluble molecules were prepared using self-made monodisperse porous poly(chloromethylstyrene-co-divinylbenzene) beads as the solid-phase matrix and ampicillin (AMP) as the template molecule. MIPs were synthesized using different template molecule: monomer: crosslinker ratios and the optimum preparation ratio were obtained by measuring adsorption. The maximum equilibrium amount of adsorption by the MIPs reached 115.62 mg/g. Scatchard analysis indicated that the MIPs contained two types of recognition sites: specific and nonspecific. Based on the adsorption kinetics, adsorption equilibrium was reached after 30 minutes. Penicillin G, amoxicillin, and sulbactam acid were used as competitive molecules to research the selective adsorption capacity of the MIPs. The imprinted material was found to have good selectivity with selectivity coefficients for penicillin G, amoxicillin, and sulbactam acid of 5.74, 6.83, and 7.25, respectively. The MIPs were used as solid-phase extraction filler, resulting in successful enrichment and separation of ampicillin residue from egg samples. Standard addition recovery experiments revealed that recovery was good with recoveries from the spiked samples ranging from 91.5 to 94.9% and relative standard deviations from 3.6 to 4.2%. The solid-phase extraction MIPs microcolumn was reused 10 times, where it maintained a recovery rate of over 80%. This work presents a sensitive, fast, and convenient method for the determination of trace ampicillin in food samples.

## 1. Introduction

Ampicillin (AMP) is a broad-spectrum penicillin used to treat infectious diseases, such as respiratory system and intestinal infections and endocarditis [1, 2]. However, excessive use of AMP can result in varying levels of residues being present in animal-based foods, which can be detrimental to consumers [3]. Current methods of detecting AMP involve high-performance liquid chromatography (HPLC) [4–6], gas chromatography-mass spectrometry (GC-MS) [7, 8], liquid chromatography-tandem mass spectrometry (LC-MS/MS) [9], and surface-enhanced Raman spectroscopy (SERS) [10–12]. However, the majority of these detection methods are used for qualitative analysis and there are few methods available for efficient quantitative analysis of trace residues [13].

Compared with other methods, molecular imprinting technology (MIPs) can produce a polymer with specific recognition ability to quickly and efficiently separate the target substance. Molecularly imprinted polymers microspheres can simulate antigen recognition by antibodies using molecular recognition holes that match the corresponding template molecules in shape, size, and functional groups [11–14]. MIPs generated using molecular imprinting technology have high selectivity and affinity for specific analytes or a group of structurally relevant compounds [15]. Conventional MIPs are synthesized by bulk polymerization, suspension polymerization, and precipitation polymerization [16–18]. However, in the preparation process, due to the use of a large amount of organic solvent, the polymerization reaction is slow (usually the preparation cycle is long), and the formed polymer has a narrow particle size distribution, which is not conducive to mass transfer. In addition, the recognition sites of the MIPs prepared by the conventional method are mostly located inside the polymer, and when the template molecules are eluted, they cannot be completely eluted, and some residues remain. As a result, the binding site is reduced and the adsorption capacity is lowered [19–22]. The emergence of surface molecular imprinting technology is expected to solve the above problems.

The surface molecular imprinting technique can be used to prepare MIPs with recognition sites on the surface. This method can effectively overcome the “embedding” defects that occur in traditional MIPs and increase the mass transfer rate [23, 24]. Surface-initiated atom transfer radical polymerization (SI-ATRP) is a free radical-polymerization technology based on molecular self-assembly that is used to prepare high-density controllable polymer brushes on the surfaces of solid matrixes [25]. This method can effectively improve imprinting efficiency, molecular recognition by the imprinted polymer, specific recognition ability, and mass transfer rate [26–28]. Concurrently, due to the involvement of water-soluble substances, MIPs preparation has always been difficult. It is important to be able prepare MIPs for water-soluble drugs and while the traditional methods of preparing MIPs have issues overcoming water phase recognition, SI-ATRP technology can effectively overcome this problem. Christian et al. [29] prepared AMP-specific recognition material using a complex process because the grinding involved in previously described techniques will destroy the recognition sites and affect imprinting. Mao et al. [30] prepared a magnetic carbon microsphere surface molecularly imprinted adsorbent by solvothermal method to identify and selectively adsorb ampicillin in milk, but the preparation process was complicated. Li et al. [31] prepared magnetic surface-imprinted material with good selectivity towards cephalexin using SI-ATRP, which was applied to detect trace residues in tap water and milk samples and displayed good enrichment.

Chloromethyl styrene resin is a functional material with a special purpose. These resin particles have good single dispersibility, are chemically stable, are easy to render hydrophilic, and have a large specific surface area [32–34]. Monodisperse chlorinated methyl styrene is a porous, highly reactive benzyl chloride that can be used as a trigger in the SI-ATRP reaction, simplifying surface modification of the matrix [35–37]. A combination of surface imprinting of monodisperse porous poly(chloromethylstyrene-co-divinylbenzene (P_VBC-DVB_) microspheres and solid phase extraction (SPE) can yield good specificity and recognition in a small column and, thus, high selectivity and separation efficiency, making it one of the most promising current technologies.

In this study, surface-imprinted MIPs that can identify water-soluble AMP molecules were prepared using SI-ATRP. MIPs was characterized by elemental analysis, thermogravimetric analysis (TGA), Fourier transform infrared spectroscopy (FT-IR), and scanning electron microscopy (SEM). The MIPs were subsequently used as an SPE adsorbent in combination with HPLC to detect trace amounts of AMP in egg samples.

## 2. Materials and Methods

### 2.1. Reagent and Instrument

AMP (99% purity), styrene (chemically pure), 4-vinylbenzyl chloride (VBC, 90% purity), azodiisobutyronitrile (analytical grade), polyvinyl alcohol (analytical grade), sodium dodecyl sulfate (SDS, analytical grade), polyvinylpyrrolidone (analytical grade), acrylamide (AM), ethylene glycol dimethacrylate (EDMA, 99% purity), *α*-bromoisobutyryl bromide, cuprous bromide (CuBr), and 2,2′-dipyridyl (Bpy) were purchased from the Aladdin reagent company (Shanghai, China). Toluene, methanol, glacial acetic acid, and tetrahydrofuran were obtained from the Tianjin Damao Chemical Reagent Factory (Tianjin, China). Penicillin G (99% purity), amoxicillin (98% purity), and sulbactam (99% purity) were purchased from Dalian Meilun reagent. Egg samples were purchased from local supermarkets.

EDMA was extracted using aqueous sodium hydroxide and distilled water and then dried with anhydrous calcium chloride. Toluene and tetrahydrofuran were distilled using sodium to remove any water. All other reagents were analytical grade.

All chromatographic tests were performed using an LC-20AT chromatographic system (Shimadzu, Japan) that included two LC-20AT pumps and an SPD-20A UV–VIS detector. Thermogravimetric analysis (TGA) was carried out using a Setsys Evolution (SETARAM, France). A scanning electron microscope was purchased from the JEOL company (JSM-7500F, Japan). FT-IR was performed on FTIR-8400S (Shimadzu, Japan). SEM was performed on JSM-7500F (JEOL, Japan). BET was performed on Brunner-Emmet-Teller measurements (NDVA-2000e, USA) An elemental analyzer was purchased from the YiLe Man element analysis system company (VarioEL III, Germany). A constant temperature water bath oscillator was purchased from the Shanghai pudong physical optics instrument plant (SHZ-C, Shanghai, China). A TG16-WS high-speed centrifuge (Centrifuge Factory, China) was used in this study and a TU-1810-type ultraviolet spectrophotometer was purchased from the Beijing general instrument Co., Ltd. (Beijing, China).

### 2.2. Preparation of Ampicillin Molecularly Imprinted Polymers

#### 2.2.1. Preparation of Monodisperse *P*_VBC-DVB_ Microspheres

Monodisperse P_VBC-DVB_ microspheres were prepared by one-step seed swelling [38, 39]. The polystyrene seed was placed in a three-neck flask and an appropriate amount of SDS solution was added. This solution was stirred while incubating in a water bath. In a dry beaker containing azodiisobutyronitrile, VBC, and DVB, the ultrasound process was performed with dibutyl phthalate, toluene, 5% polyvinyl alcohol, 0.2% SDS, and distilled water. Following ultrasonic emulsification, the solution was rapidly transferred to a three-neck bottle, and swelling occurred at 25°C for 24 hours and then at 70°C in a nitrogen atmosphere for 24 hours. The product was extracted with a Soxhlet and the P_VBC-DVB_ microspheres were obtained by drying with a vacuum.

#### 2.2.2. Preparation of Ampicillin Molecularly Imprinted Polymer Microspheres

Template AMP (1 mmol) was placed in a 100 mL round-bottom flask with 40 mL water:acetonitrile (3:5, V/V) as solvent, 4 mmol AM as functional monomer, and 15 mmol EDMA as crosslinker and prepolymerized for 4 hours at 40°C to form a prepolymerized complex solution. P_VBC-DVB_ microspheres (2 g) were placed in another 100 mL round-bottom flask and 0.1952 g Bpy and 0.0284 g CuBr were added to a self-made N_2_ circulation device. After 30 minutes, the above prepolymerized composite solution was quickly transferred to a round-bottom flask containing P_VBC-DVB_ microspheres. The polymerization reaction was performed at room temperature for 20 hours. After the reaction, the solution was washed with a large amount of deionized water and EDTA solution and then dried. The dried polymer was extracted with methanol: glacial acetic acid (9:1, V/V) and a Soxhlet for 24 hours followed by pure methanol for 8 hours and then dried at 50°C in a vacuum to obtain AMP MIPs microspheres.

The preparation of nonimprinted polymers (NIPs) was the same as above except that no template molecule was added.

### 2.3. Adsorption Experiment

The adsorption isotherm was determined by adding 10 mg of MIPs to 10 mL of acetonitrile 0.1 mol/L aqueous NaOH (9:1, V / V), wherein the AMP concentration was 1-10 mmol/L. The mixture was shaken at room temperature for 10 hours. The mixture was separated by centrifugation and the solution was filtered through a 0.45*μ*m membrane. The concentration of the filtered solution was measured with a UV spectrophotometer. The same procedure was performed for the NIPs and all tests were performed in triplicate.

The adsorption capacity (Q) is calculated according to

(1) $$\begin{array}{c} Q=\frac{\left(C_{0}-C_{t}\right)Vm}{M} \end{array}$$

where C_0_ (mmol/L) is the initial concentration of AMP, C_t_ (mmol/L) is the concentration of equilibrium AMP, Vm (mL) is the total volume of the adsorbed mixture, and M(g) is the mass of the MIPs.

The kinetics of AMP on MIPs was investigated at room temperature by the addition of 10 mg of MIPs in 10 mL of 10 mmol/L AMP solution. The mixture was shaken on a shaker for various times and centrifuged at 4000 rpm for 2 minutes and then filtered to perform UV analysis at 254 nm. Perform the same experimental steps for NIPs.

Penicillin G, amoxicillin, and sulbactam acid were chosen as competitors to estimate the selectivity of MIPs for AMP. Disperse 10 mg of MIPs in 10 mL of 0.1 mol/L NaOH-acetonitrile (1:9, V/V) solution containing Penicillin G, amoxicillin, and sulbactam acid at an initial concentration of 10 mmol/L. After adsorption, UV spectrometry A photometer measures the equilibrium concentration of each analyte.

The values of K_D_ and IF are the basic measures of imprinted polymers [40], which are calculated by (2) and (3). Larger K_D_ and IF values mean that the polymer has excellent adsorption affinity.

(2) KD=QCt

(3) IF=kMIPskNIPs

where Q (mmol / g) is the binding amount of MIPs and NIPs; Ct (mmol / L) is the equilibrium concentration of AMP; K_D_ (mL / g) is the partition coefficient; k_MIPss_ and K_NIPs_ are the partition coefficients of MIPss and NIPs. IF stands for the imprinting factor of MIPs.

### 2.4. Actual Sample Determination

Egg sample (50 g) was mixed with 250 mL of ethanol:water (6:4, V/V), incubated on an oscillator for extraction for 30 min, and then centrifuged. The resulting supernatant was mixed with ethanol:water (1:1, V/V) to a final volume of 100 mL and filtered with a 0.22 *μ*m filter membrane. The filtrate was sealed and refrigerated at 3°C. The final concentrations were 5, 20, and 50 ng·g^−1^.

For the MIPs-SPE [41], NIPs-SPE, and C_18_-SPE, 1 mL of the above solution was used. AMP adsorbed on the cartridge was eluted using methanol:glacial acetic acid (8:2, v/v). The extract was blown dry with N_2_ and the mobile phase was brought to a constant volume. The concentration of AMP was measured by HPLC and each addition level was measured 3 times.

The chromatographic conditions consisted of a Diamonsil C_18_ column (150mm × 4.6mm, 5 *μ*m), a mobile phase of 40:60 (V/V) acetonitrile-water with 0.1% acetic acid, an injection volume of 10 *μ*L, and a detection wavelength of 230 nm.

## 3. Results and Discussion

### 3.1. Preparation of MIPs

P_VBC-DVB_ microspheres were prepared using the “one-step seed swelling method.” The first step was to obtain micron-sized monodisperse styrene microspheres with a lower molecular weight by dispersing polymerization in an organic medium and then using the microspheres as seed liquid. Swelling polymerization was performed directly in the aqueous phase to obtain monodisperse P_VBC-DVB_ microspheres. Microspheres have the advantages of good hydrophilicity and easy surface modification. When preparing MIPs using SI-ATRP technology, modification of the surface initiator group can be omitted and, thus, preparation can be simplified.

**Figure 1:**
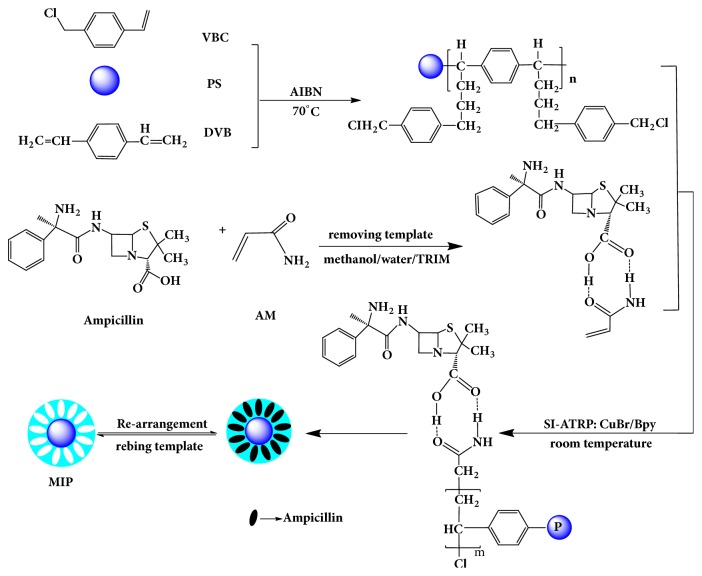
Synthetic route of surface-imprinted polymers (MIPs) microspheres.

Preparation of AMP MIPs microspheres used CuBr/Bpy as a catalytic system, free radical-initiated polymerization in a water:acetonitrile mixed solution, and a free radical-initiated reaction. The prepolymerized complex formed by the functional monomer AM and imprinted molecule AMP, and EDMA crosslinking agent were grafted onto the surface of the P_VBC-DVB_ microspheres. Then the template molecules were removed and AMP MIPs microspheres were obtained. The graft density and adsorption properties of the imprinted materials were controlled by altering the monomer:template:crosslinker ratio. (Figure 1)

### 3.2. Characterization of MIPs

#### 3.2.1. Elemental Analysis

The polymers of P_VBC-DVB_ and MIPs were characterized by using elemental analysis. The elemental analysis data are listed in (Table 1). Compared with P_VBC-DVB_, the contents of the C and H elements were significantly increased. This evidence indicates that the crosslinker EDMA has been successfully grafted to the P_VBC-DVB_ surface.

#### 3.2.2. Thermogravimetric Analysis

**Figure 2:**
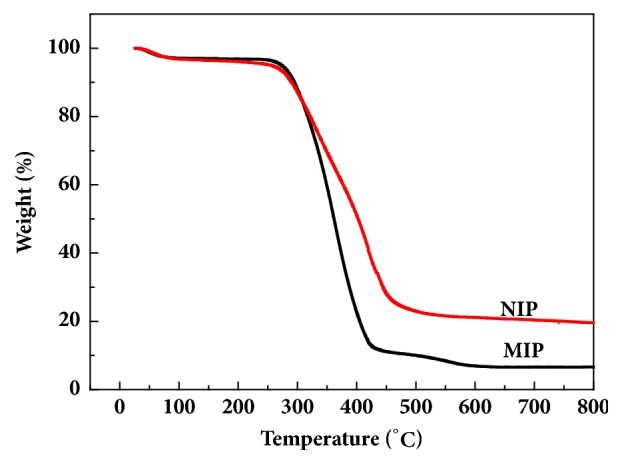
Thermogravimetric curves of P_VBC-DVB_, MIP, and nonimprinted polymers (NIPs).

Thermogravimetric analysis results for MIPs and NIPs are shown in (Figure 2) The two polymers lose about 2.8% at 25°C to 110°C, and the main loss component is water. The polymer decomposes rapidly from 300°C to 500°C, and the MIPs decompose rapidly at 237°C to 470°C, and the weight loss is 77.8%. The NIPs decompose the fastest at 224°C to 494°C, and the weight loss is 76.88%. The difference may be due to the interaction of template molecules and functional monomers in MIPs affecting the weight loss characteristics of polymers.

#### 3.2.3. FT-IR Characterization of *P*_VBC-DVB_ Microspheres and MIPs

**Figure 3:**
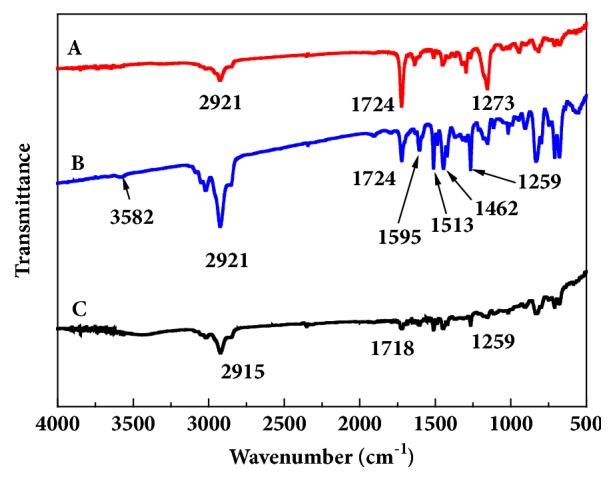
FT-IR spectra of (A) P_VBC-DVB_ microspheres, (B) uneluted MIPs, and (C) eluted MIPs.

It can be seen from the figure that the strong absorption peak at 1724 cm^−1^ and 1259 cm^−1^ is the stretching vibration of -C=O in the carboxyl group and the symmetric vibration of -C-O in the EDMA ester group. It is indicated that AM and EDMA successfully polymerize on the surface of polystyrene microspheres in the presence of initiator AIBA [42] (Figure 3(A)). The carbonyl -C=O vibration peak of the *β*-lactam ring of the AMP molecular structure appeared at 1770 cm^−1^ in (Figure 3(B)), and new peak positions appeared at 1595 cm^−1^, 1513 cm^−1^, and 1462 cm^−1^. These peaks are a characteristic peak of the benzene ring in the AMP molecular structure, indicating the presence of AMP in the imprinted material [43]. These peaks are not seen in (Figure 3(C)), which is the elution of the imprinted material leaving only holes similar in structure to the template molecule and without the template molecule AMP.

#### 3.2.4. Electron Microscopy Analysis and Physisorption Measurements

**Figure 4:**
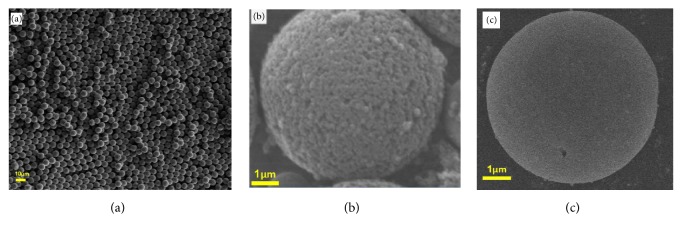
Scanning electron micrographs of (a) P_VBC-DVB_ microspheres, (b) MIPs, and (c) NIPs.

SEM was used to observe the morphological structure of P_VBC-DVB_ microspheres (a), MIPs (b), and NIPs (c). As shown in (Figure 4(a)), the P_VBC-DVB_ microspheres prepared by the “one-step seed swelling method” were uniform in size and uniform in dispersion and had good monodispersity. After imprinting, the surface of MIPs (Figure 4(b)) is rough and uneven, with morphological features and uniformly distributed pores, which facilitates the adsorption of molecules on mass transfer. The morphological characteristics of NIPs (Figure 4(c)) are not as obvious as MIPs, and the surface pore size is almost absent. The comparison results show that the roughness of the surface of the imprinted polymer increases.

Table 2 shows the specific surface area, pore volume, and average pore size of MIPs and NIPs. As can be seen from Table 2, MIPs have larger specific surface area, pore volume, and average pore size than NIPs. The results showed that the different adsorption properties of MIPs and NIPs could not be completely attributed to the difference in morphology but also related to the imprinting process that produced specific recognition sites [44].

### 3.3. Binding Properties of the MIPs and NIPs

#### 3.3.1. Equilibrium Adsorption Curve of Ampicillin with Different MIPs

**Figure 5:**
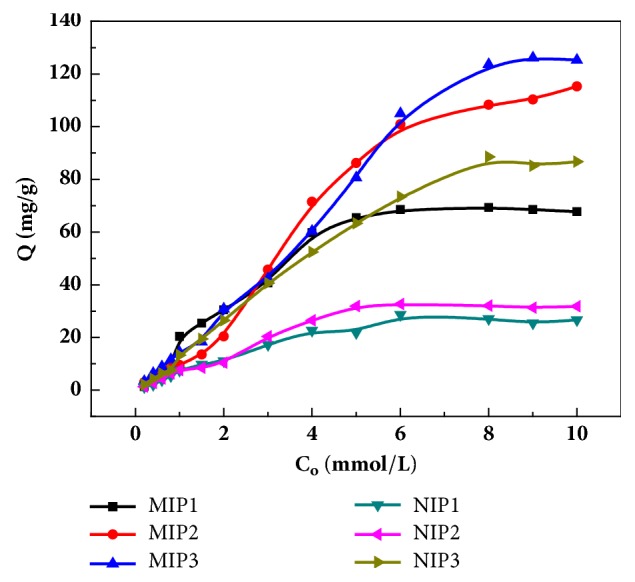
Equilibrium adsorption curves of ampicillin with different MIPs and NIPs.

Three groups of imprinted polymers were prepared with fixed template:functional monomer:crosslinker ratios of 1:3:20, 1:4:20, and 1:5:20 and their equilibrium adsorption curves were generated (Figure 5). As can be seen from (Figure 5), as the monomer concentration increased, the amount of imprinted and nonimprinted polymer adsorbed also increased. The difference in the adsorption amounts between MIP3 and NIP3 were small because nonspecific adsorption strengthened as the monomer concentration increased, which reduced the specific adsorption performance of the MIPs. In Figure 5, the adsorptions of MIP1 and NIP1 were relatively low and the difference between them very small because the monomer concentration was too low, rendering it difficult to form effective recognition sites and spatial structures in the polymer and resulting in a low adsorption and poor selectivity. As the AMP concentration increased, as in MIP2, the adsorption capacity of the MIPs also increased and was much larger than that of NIP2. It can be seen that MIP achieved the best recognition when the template molecule:monomer:crosslinker ratio was 1:4:20. The maximum equilibrium adsorption capacity of MIP2 was about 115.62 mg/g. The maximum equilibrium adsorption capacity of NIP2 was only 33.40 mg/g, which was far less than that of MIP2. This is because for the monodisperse imprinted matrix P_VBC-DVB_ microspheres, the surface recognition sites were more evenly distributed and the surface recognition sites formed were more conducive to the enrichment of template molecules, which are jointly affected by both specific and non-specific adsorption. MIP2 exhibited the strongest adsorption performance.

**Figure 6:**
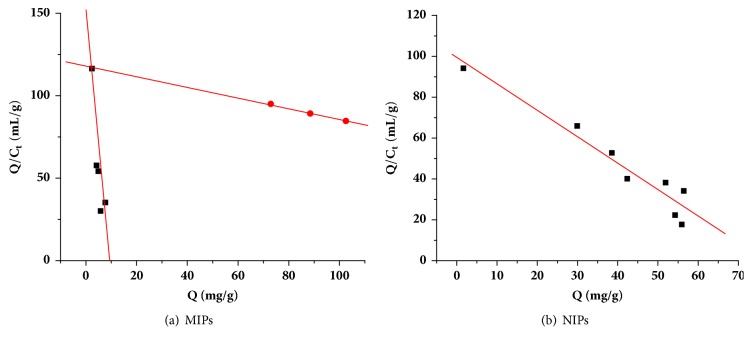
Scatchard fitting curves for (a) MIPs and (b) NIPs microspheres.

Scatchard plots [45, 46] were created for Q/c and Q. As shown in the MIP graph in Figure 6, there were two distinctly good linear relationships for AMP adsorption on the MIPs, indicating that there were two different types of binding sites. After fitting them separately and calculating the performance of the two types of binding, the maximum adsorption capacities were determined to be 359.08 and 9.04 mg/g. The adsorption capacity of the MIP was higher than for other materials. K_Ds_ were 3.04 and 0.072 mg/L.

#### 3.3.2. Kinetic Adsorption

**Figure 7:**
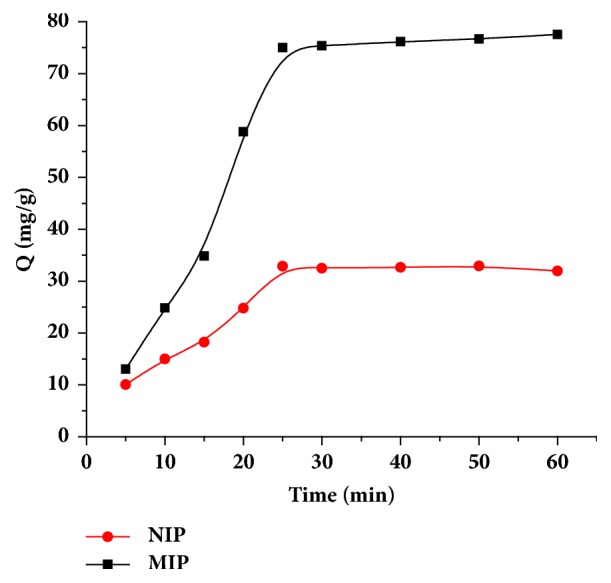
Dynamic adsorption curves.

Figure 7 shows the kinetics of MIPs and NIPs adsorption. It can be seen from the figure that the adsorption rates of MIPs and NIPs were very fast during the first 20 minutes and the adsorption equilibrium was reached in about 30 minutes. Traditional bulk MIPs has a slow adsorption rate of 10-24 h. This is because the surface imprinting method forms a recognition hole with recognition sites on the surface of the polymer, where uniform distribution facilitates entry of the imprinting molecule into the recognition hole and quick spreading on the inside of the imprinting hole to achieve adsorption equilibrium.

#### 3.3.3. Adsorption Selectivity

**Figure 8:**
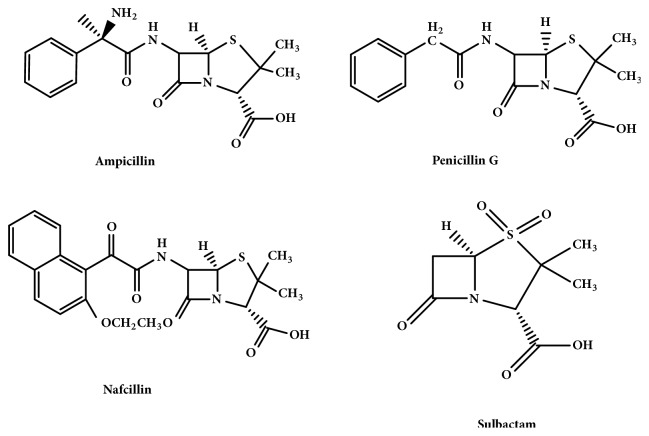
The structures of ampicillin, penicillin G, nafcillin, and sulbactam.

Three penicillin antibiotics with structures similar to AMP (Figure 8) were used to study the specific recognition ability of the MIPs [47].

**Figure 9:**
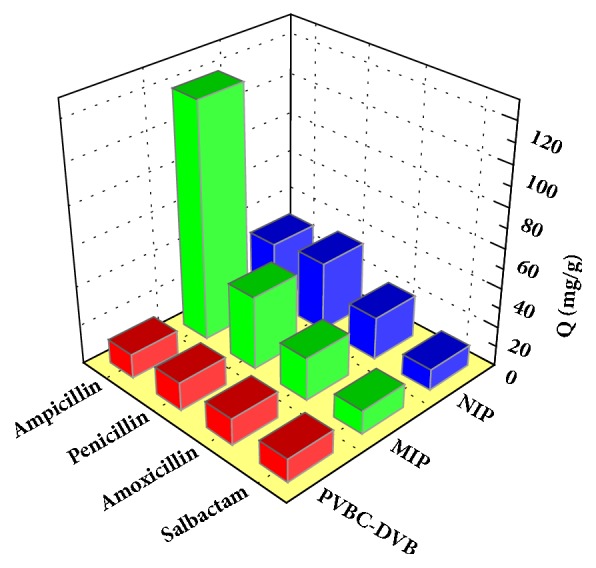
Selective adsorptions.

From Figure 9, analysis of the adsorption of the four antibiotics by P_VBC-DVB_ microspheres matrix, MIPs, and NIPs revealed that MIPs had the largest adsorption capacity and best selectivity for AMP. The differences in the adsorption capacity of NIPs for the four antibiotics were very small because recognition of the molecules by NIPs was controlled by nonspecific adsorption and determined based on the strength of the interactions between the monomer and antibiotic molecules. Recognition of antibiotic molecules by MIPs was influenced by the functional groups and size and spatial structure of the cavities imprinted on the surface.

It can be seen that the relative selectivity coefficient of the MIP for the imprinting molecule AMP was 4.6, showing that the AMP MIPs microspheres had good molecular recognition. Compared to the P_VBC-DVB_ matrix and NIPs, MIPs displayed corresponding recognition. The MIP had recognition pores the same as the template molecule in terms of size and spatial structure and exhibited specific adsorption of the template molecule. The selectivity coefficients of MIPs for the three structural analogs were 5.74, 6.83, and 7.25, respectively, while the selectivity coefficients of NIPs and P_VBC-DVB_ microsphere substrates to the structural analogs were between 0.73 and 1.63. Therefore, the MIPs exhibited better recognition selectivity and AMP MIPs microspheres had good molecular affinity and molecular recognition capabilities. (Table 3)

### 3.4. Reuse Performance of MIPs-SPE

**Figure 10:**
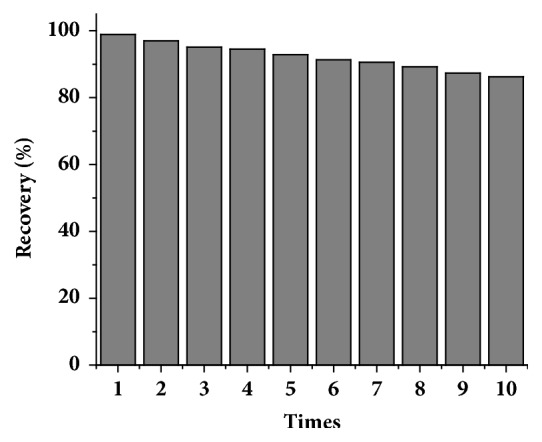
MIPs-SPE cycle performance.

The ability for MIP to be used repeatedly is also an important criterion when evaluating the performance of MIPs. To examine the reusability of MIPs-SPE, 10 recovery experiments were performed on the same cartridge. As shown in (Figure 10), when the MIPs-SPE cartridge was repeatedly used 10 times, it had a recovery rate of over 80%, indicating the repeated use performance was good. Therefore, the MIP was a good reusable SPE sorbent, which greatly reduced experimental cost.

### 3.5. Molecularly Imprinted Solid-Phase Extraction of AMP from Spiked Samples

**Figure 11:**
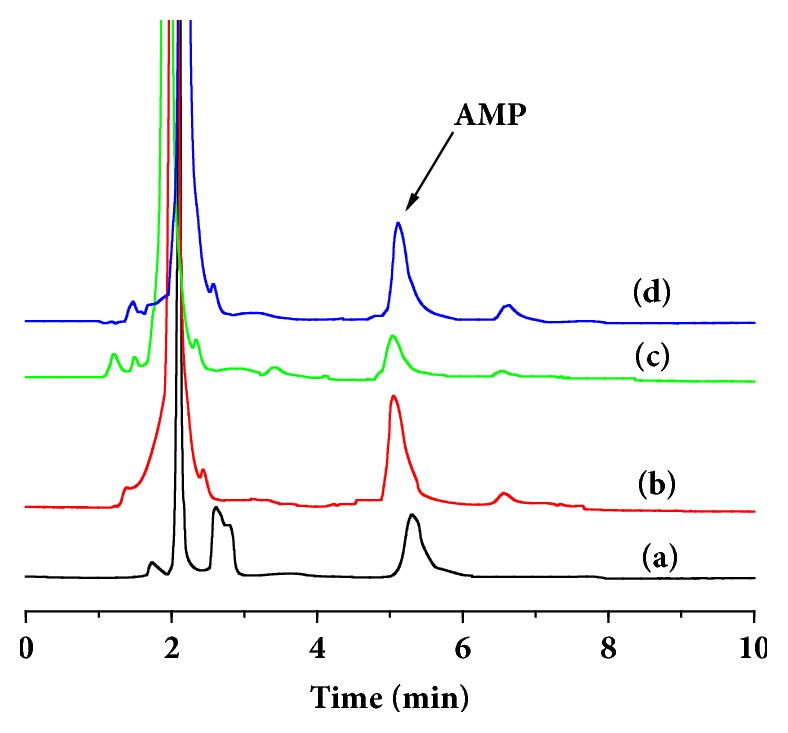
Chromatograms of egg samples processed on different solid phase extraction (SPE) columns: (a) C_18_-SPE cartridge, (b) MIPs-SPE cartridge, (c) NIPs-SPE cartridge, and (d) standard solution.

Figure 11(d) shows the chromatograms of mixed standard solutions of AMP and penicillin G. The peaks corresponding to AMP and penicillin G occurred at 4.91 and 6.13 min, respectively. The residual amount of AMP in the blank egg sample was approximately 18.87 *μ*g·g^−1^, which did not exceed the national minimum limit. The linear equation of the AMP standard solution in the range of 5-10000 ng/g was Y=10.494 X-758.08 and the correlation coefficient was R=0.9995. C_18_, MIPs, and NIPs were used as SPE matrixes under optimized experimental conditions and the content of AMP in eggs was measured by HPLC. For the MIP-SPE, the average recoveries were 91.5, 92.4, and 94.9% and the relative standard deviations were 3.6, 4.1, and 4.2%. The specific values are listed in Table 4. It can be clearly seen that MIPs-SPE treatment had an enrichment and purification effect on the samples, resulting in a higher extraction recovery rate than when using NIP-SPE or C_18_-SPE. The results show that MIPs-SPE facilitates the detection of trace AMP in complex samples.

## 4. Conclusions

In this study, MIPs microspheres with the ability to specifically recognize water-soluble molecules were prepared using SI-ATRP technology and the specificity of recognition by these MIPs was studied. In adsorption tests, the MIPs displayed excellent recognition and AMP selectivity. The MIPs displayed a lower binding capacity to the AMP structural analogues of penicillin G, nafcillin, and sulbactam. The generated material can be successfully used as SPE adsorbent for HPLC separation, enrichment, and detection of trace AMP in food samples. When MIPs was used as SPE filler to measure the remaining trace amounts of AMP in egg, the recovery rate was found to be good, ranging between 91.5 and 94.9% with relative standard deviations of 3.6 to 4.2%. Repeated use of the MIPs as SPE material 10 times yielded a recovery rate above 85%, indicating the MIP had good stability and repeatability with a good rich set and high recovery. Overall, this study shows that MIPs is an ideal material for enrichment and detection of trace AMP residue and provides means for quantitatively and qualitatively analyzing AMP in other complex substrate samples. In addition, this research method provides an important reference for further monitoring and research to improve food safety.

## Figures and Tables

**Table 1:** Elemental analysis of imprinted materials.

Material	Elemental composition (%,w/w)		
	C	N	H
P_VBC-DVB_	77.96	0.543	6.951
MIPs	80.98	0.649	6.940

**Table 2:** Comparison of MIPs and NIPs from nitrogen adsorption-desorption analysis.

Sample	Surface Area /(m^2^•g^−1^)	Pore Volume /(cm^3^•g^−1^)	Average Pore Size /(nm)
MIPs	95.26	0.247	20.76
NIPs	68.39	0.105	12.32

**Table 3:** Selective coefficients of MIPs, NIPs, and P_VBC-DVB_.

Analyte	C_e_ (mmol/L)			Q (mmol/g)			K_D_ (L/g×10^−3^)			IF			*β*
	P_VBC-DVB_	MIPs	NIPs	P_VBC-DVB_	MIPs	NIPs	P_VBC-DVB_	MIPs	NIPs	P_VBC-DVB_	MIPs	NIPs	
Ampicillin	9.59	6.80	9.07	0.041	0.320	0.093	0.0043	0.0471	0.0103	—	—	—	4.6
peillinG	9.60	9.25	9.28	0.040	0.076	0.072	0.0042	0.0082	0.0078	1.02	5.74	1.32	1.1
amoxicillin	9.88	9.56	9.60	0.035	0.066	0.062	0.0035	0.0069	0.0065	1.23	6.83	1.58	1.1
Salbactam	9.48	9.43	9.44	0.056	0.061	0.059	0.0059	0.0065	0.0063	0.73	7.25	1.63	1.0

*∗*C_o_=10 mmol/L.

**Table 4:** Standard addition of ampicillin and relative standard deviations of ampicillin recovery from egg samples.

Adsorbent	Amount added (ng·g-1)	Recovery (R/%)			Mean recovery ($\bar{R}$/ %)	RSD/%
		1	2	3		
MIP-SPE	5	90.6	99.1	95.1	94.9	4.2
	20	92.7	87.4	94.3	91.5	3.6
	50	91.1	97.0	89.2	92.4	4.1
NIP-SPE	5	12.3	22.5	25.6	20.2	6.9
	20	17.8	16.1	8.3	14.1	5.1
	50	26.7	26.7	27.6	27.0	0.5
C18-SPE	5	77.3	78.3	73.9	76.5	2.3
	20	91.5	87.7	93.8	91.0	3.1
	50	92.5	97.1	92.7	94.1	2.6

## Data Availability

The data used to support the findings of this study are included within the article.
